# Does Chronic Sleep Fragmentation Lead to Alzheimer's Disease in Young Wild-Type Mice?

**DOI:** 10.3389/fnagi.2021.759983

**Published:** 2021-12-21

**Authors:** Li Ba, Lifang Huang, Ziyu He, Saiyue Deng, Yi Xie, Min Zhang, Cornelius Jacob, Emanuele Antonecchia, Yuqing Liu, Wenchang Xiao, Qingguo Xie, Zhili Huang, Chenju Yi, Nicola D'Ascenzo, Fengfei Ding

**Affiliations:** ^1^Department of Neurology, Tongji Hospital, Tongji Medical College, Huazhong University of Science and Technology, Wuhan, China; ^2^Department of Biomedical Engineering, School of Life Science and Technology, Huazhong University of Science and Technology, Wuhan, China; ^3^Department of Medical Physics and Engineering, Istituto Neurologico Mediterraneo Neuromed Istituto di Ricovero e Cura a Carattere Scientifico (I.R.C.C.S.), Pozzilli, Italy; ^4^Department of Electronic Engineering and Information Science, University of Science and Technology of China, Hefei, China; ^5^Department of Pharmacology, Shanghai Medical College, Fudan University, Shanghai, China; ^6^Research Centre, The Seventh Affiliated Hospital of Sun Yat-sen University, Shenzhen, China

**Keywords:** sleep fragmentation, Alzheimer's disease, stress, F-18-fluorodeoxyglucose-positron emission tomography (^18^F-FDG-PET), neuroinflammation, tau, amyloid-β

## Abstract

Chronic sleep insufficiency is becoming a common issue in the young population nowadays, mostly due to life habits and work stress. Studies in animal models of neurological diseases reported that it would accelerate neurodegeneration progression and exacerbate interstitial metabolic waste accumulation in the brain. In this paper, we study whether chronic sleep insufficiency leads to neurodegenerative diseases in young wild-type animals without a genetic pre-disposition. To this aim, we modeled chronic sleep fragmentation (SF) in young wild-type mice. We detected pathological hyperphosphorylated-tau (Ser396/Tau5) and gliosis in the SF hippocampus. ^18^F-labeled fluorodeoxyglucose positron emission tomography scan (^18^F-FDG-PET) further revealed a significant increase in brain glucose metabolism, especially in the hypothalamus, hippocampus and amygdala. Hippocampal RNAseq indicated that immunological and inflammatory pathways were significantly altered in 1.5-month SF mice. More interestingly, differential expression gene lists from stress mouse models showed differential expression patterns between 1.5-month SF and control mice, while Alzheimer's disease, normal aging, and APOEε4 mutation mouse models did not exhibit any significant pattern. In summary, 1.5-month sleep fragmentation could generate AD-like pathological changes including tauopathy and gliosis, mainly linked to stress, as the incremented glucose metabolism observed with PET imaging suggested. Further investigation will show whether SF could eventually lead to chronic neurodegeneration if the stress condition is prolonged in time.

## Introduction

Sleep is a highly conserved physiological phenomenon among mammals and important for multiple physiological processes, including cognitive function, immune function, and hormone release (Irwin, 2015; Krause et al., 2017). During a normal night sleep, non-rapid eye movement (NREM) and rapid eye movement (REM) sleep alternately occur for 5–6 episodes in humans. Both sleep stages are important for learning and memory consolidation. It has been shown that the risk of developing Alzheimer's disease and the prevalence of all-cause dementia increases with sleep disorders (Shi et al., 2018). In clinical observation, sleep disturbance is often present years before the symptomatic stages of neurodegenerative diseases and becomes more severe along with the disease progression (Guarnieri et al., 2012; Irwin and Vitiello, 2019). In the APP/PS1 animal model of Alzheimer's disease, partial sleep deprivation could accelerate Aβ plaques depositions and cognition impairment (Wang et al., 2021). All these important pieces of evidence pointed to the hypothesis that, sleep disturbance might be a key etiology of neurodegenerative diseases, especially AD. This hypothesis was mechanism-wise strongly supported by the recent discovery of a glia-based system, called the Glymphatic System, which manages the convection flows through brain parenchymal and drains the neurotoxic substances out of the brain (Iliff et al., 2012). In young wild-type mice, much higher efficiency of cerebrospinal fluid (CSF) -interstitial fluid (ISF) exchange and drainage was found occurring in the sleep phase *in vivo* (Iliff et al., 2012; Xie et al., 2013). Several follow-up studies reported the functional failure of the Glymphatic System was evident in neurodegenerative disease animal models, including i.e., Alzheimer's disease (AD) and Parkinson's disease (PD) (Rasmussen et al., 2018; Nedergaard and Goldman, 2020). These studies proposed that sleep disturbance could dampen interstitial space substance clearance, resulting in neurodegeneration due to excessive neurotoxic protein accumulation (Ross and Poirier, 2004). Neuronal metabolic waste, such as soluble Aβ and adenosine, exhibited circadian rhythms and accumulated in brain interstitial space after acute sleep deprivation (Kang et al., 2009; Roh et al., 2012; Wu et al., 2016; Peng et al., 2020). Based on these results, we were wondering if a young and healthy brain could be turned into a neurodegenerative brain under continuous sleep disturbance. It is a frightening hypothesis especially for people who have long-term inevitable night shifts and sleep insufficiency. It is a serious issue if we consider that chronic sleep insufficiency is becoming a common issue in young populations nowadays, mostly due to life habits and work stress. A study conducted in the population of university students reported that 40% of students with an average age of around 20-year-old had smartphone addiction and these students exhibited significantly poorer subjective sleep quality and more severe daytime dysfunction than the ones without smartphone addiction (Lane et al., 2021). In young to middle-aged medical workers, more than 50% had abnormal daytime sleepiness and poor sleep quality (Carvalho et al., 2021).

So far, there is no direct answer to the key scientific question of whether chronic sleep insufficiency could lead to neurodegeneration even in the absence of factors involving genetic pre-disposition and senescence. In our previous study, we reported chronic sleep fragmentation (SF) interventions induced AD-like pathology in young wild-type C57BL/6 mice. We found that 1.5-month SF treatment resulted in cognitive impairment, intracellular Aβ_1−42_ accumulation, gliosis, and dysfunction of the endosomal-autophagosome-lysosomal (EAL) pathway(Xie et al., 2020a). Takahashi et al. observed in human autopsy samples that intracellular Aβ_1−42_ accumulation was only seen at pre-symptomatic or early stage of AD but was absent at symptomatic stage (Takahashi et al., 2017). It has not been tested yet if pathological tau-aggregation, another even more important hallmark of AD pathogenesis (Wang and Mandelkow, 2016), occurs in chronic SF brain. Meanwhile, it has been reported glucose metabolic disorder appears before symptomatic AD in APP/PS1 mice. ^18^F-labeled fluorodeoxyglucose positron emission tomography scan (^18^F-FDG-PET) scan revealed that glucose utilization increases in multiple brain regions of APP/PS1 Tg mice at 2 and 3.5 months (pre-symptomatic stage) (Li et al., 2016). So far, no study has tested glucose metabolism in chronic sleep fragmentation brain in young wild-type mice. Based on our scientific question and current evidence, we hypothesized that chronic sleep fragmentation could probably initiate preceding pathological processes for neurodegeneration even in a young healthy brain.

In the current study, we further detected the intracellular deposition of pathological hyperphosphorylated tau, gliosis with immunohistochemistry, and western blot in 1.5-month chronic SF mice in comparison with normal young wild-type mice. We also evaluated the brain glucose metabolism with ^18^F-FDG-PET and conducted hippocampal transcriptome mapping with RNA sequencing to better understand the pathological processes induced by chronic sleep fragmentation.

## Materials and Methods

### Animals

The study involved 2–3-month-old wild-type male C57BL/6J mice obtained from the Hubei Research Center for Laboratory Animals (Hubei, China) which were used for experiments. All animal procedures were approved by the Institutional Animal Care and Use Committee of Tongji Hospital, Tongji Medical College, Huazhong University of Science and Technology.

### Chronic Sleep Fragmentation Modeling

**Figure 1:**
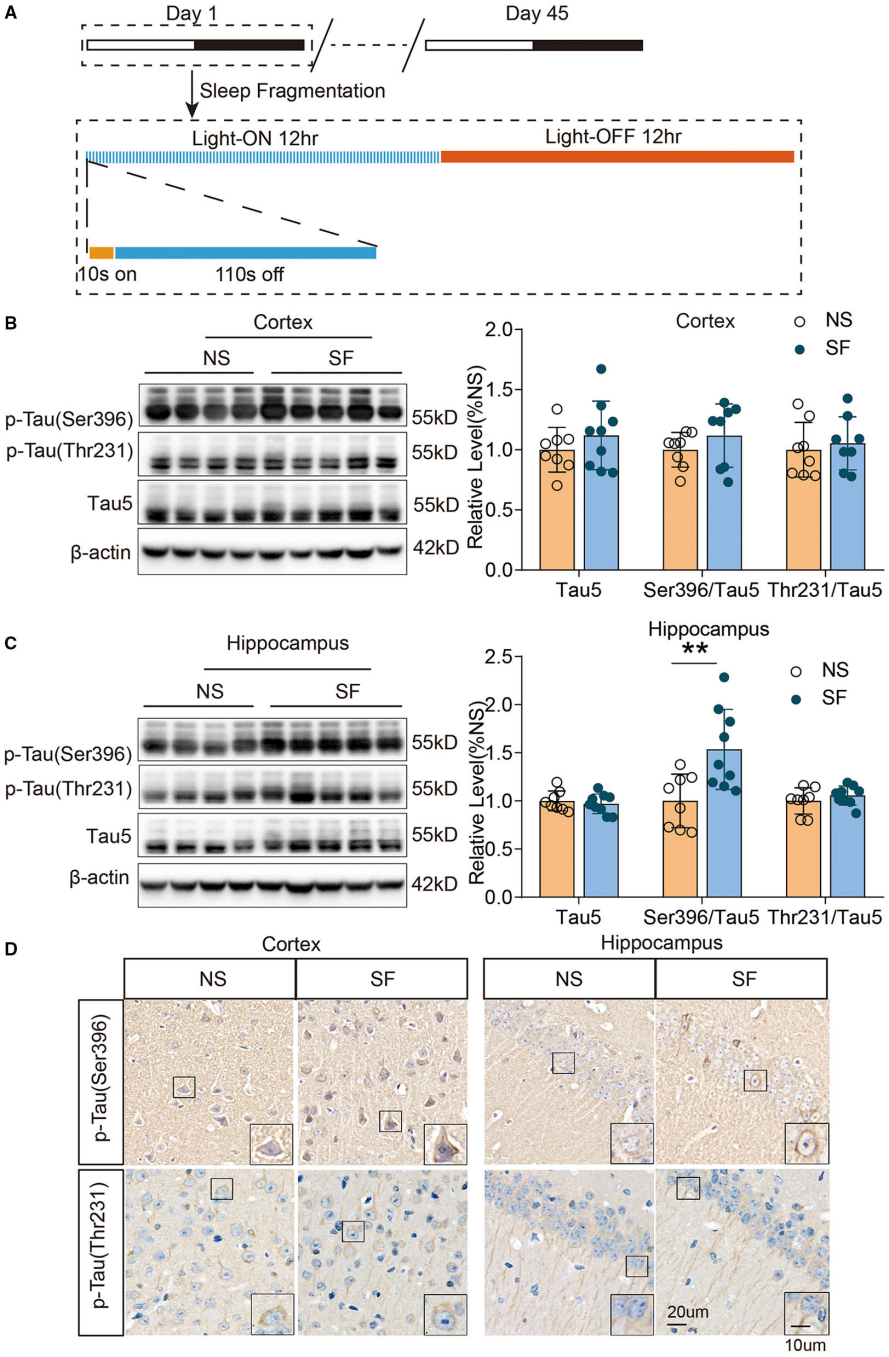
Chronic SF increases pathologically phosphorylated tau (Ser396) in young wild-type mice hippocampus. **(A)** The schematic figure of the experimental design procedure, indicating the timing of the SF model. **(B,C)** Western blotting and quantitative density of expression of p-tau Ser396, p-tau Thr231, and tau5 in the cortex **(B)** and hippocampus **(C)** show an increase of p-tau Ser396 in SF hippocampus. β-actin was used as loading control. **(D)** Representative immunohistochemistry images of phosphorylated tau (p-tau Ser396 and p-tau Thr231) in the cortex and hippocampus of SF and NS group. Scale Bar = 20 μm. Local enlarged images were presented in the boxes. Scale Bar = 10 μm. *n* = 8 for NS and *n* = 9 for SF group. **P < 0.01.

Animals were randomly divided into a chronic SF group and a normal sleep (NS) group with 11–12 mice in each group. Both populations were kept in a 12-h light-dark cycle (8:00 a.m.−8:00 p.m. light-ON, 8:00 p.m.−8:00 a.m. light-OFF) with free access to food and water. Following the procedure described previously (Xie et al., 2020a), chronic SF model cages were secured on an orbital rotor, vibrating at 110 rpm and with a repetitive cycle of 10 s-on, 110 s-off, during the light-ON phase (Figure 1A). This chronic SF procedure was performed continuously for 1.5 months (45 days). And the NS group cages were placed in the same room as the SF cages, to keep the surrounding environment and labor effects identical (Xie et al., 2020b).

### ^18^F-FDG-PET Scan

Both SF and NS mice were kept fasting for 24 h prior to the scanning. On the experimentation day, mice were anesthetized by inhalation of 2% isoflurane. Approximately 7 μCi/g 2-[^18^F]FDG diluted in a 0.1 ml solution were injected intraperitoneally. The average total dose per mouse was ~200 μCi. Then, 60 min after injection, a 10-min-long PET/CT scan (D180, RAYCAN, China) was performed. An OSEM-3D-PSF algorithm including random and attenuation corrections was adopted for image reconstruction. The CT scan consisted of 400 angular projections. CT data were reconstructed using a cone-beam algorithm for visualizing the skull structure. The voxels of the reconstructed PET and CT images had a size of 0.5 × 0.5 × 0.5 mm and 0.156 × 0.156 × 0.088 mm, respectively. The PET and CT images were co-registered with a mutual information method. Finally, the brain of the mouse was segmented with a threshold-based algorithm by using the standardized uptake value (SUV) of the PET image, followed by a topological closure. The segmented brain images were co-registered with a mouse brain atlas (Ma et al., 2005; D'Ascenzo et al., 2020) by using a mutual information method, and 19 brain regions were identified, as reported in Table 1. Finally, the SUV of each mouse was calculated both in the entire brain and in each of the segmented brain regions separately.

**Table 1:** ^18^F-FDG uptake per brain region in mice.

**Brain region**	**Average SUV in NS (g/cc)**	**Average SUV in SF (g/cc)**	**Percentage difference in SF**	***P*-value**
			**(SF-NS)/NS,%**	
Whole brain	1.47 ± 0.17	2.12 ± 0.44	44%	0.0154^*^
RSTR	1.77 ± 0.25	2.23 ± 0.40	26%	0.0627
LSTR	1.84 ± 0.26	2.35 ± 0.54	28%	0.0890
CTX	1.55 ± 0.14	2.05 ± 0.22	32%	0.0025^*^
RHIP	1.45 ± 0.18	2.27 ± 0.61	57%	0.0381^*^
LHIP	1.44 ± 0.20	2.27 ± 0.74	58%	0.0642
THA	1.46 ± 0.21	2.25 ± 0.77	54%	0.0824
CB	1.49 ± 0.20	2.36 ± 0.70	58%	0.0502
BFS	1.45 ± 0.25	1.79 ± 0.36	23%	0.1205
HYP	1.04 ± 0.13	1.57 ± 0.38	51%	0.0182^*^
RAMY	1.16 ± 0.14	1.63 ± 0.20	41%	0.0023^*^
LAMY	1.23 ± 0.18	1.97 ± 0.42	60%	0.0063^*^
BS	1.21 ± 0.21	2.01 ± 0.59	66%	0.0204^*^
CG	1.65 ± 0.25	2.61 ± 0.98	58%	0.0925
SC	1.62 ± 0.22	2.47 ± 0.83	52%	0.0829
OLF	1.39 ± 0.24	1.88 ± 0.47	35%	0.0662
RMID	1.37 ± 0.24	2.27 ± 0.84	66%	0.0742
LMID	1.42 ± 0.28	2.48 ± 0.97	75%	0.0695
LIC	1.68 ± 0.27	2.70± 0.95	61%	0.0735
RIC	1.63 ± 0.26	2.58 ± 1.00	58%	0.1023

SUV, standard uptake value; NS, normal sleep; SF, sleep fragmentation; RSTR, right striatum; LSTR, left striatum; CTX, cortex; RHIP, right hippocampus; LHIP, left hippocampus; THA, thalamus; CB, cerebellum; BFS, basal forebrain/septum; HYP, hypothalamus; RAMY, right amygdala; LAMY, left amygdala; BS, brain stem; CG, central gray; SC, superior colliculi; OLF, olfactory bulb; RMID, right midbrain; LMID, left midbrain; LIC, left inferior colliculus; RIC, right inferior colliculus; *P < 0.05.

### Tissue Preparation

The mice (*n* = 8 for NS and *n* = 9 for SF group) were sacrificed by decapitation and the brains were extracted. Brains were quickly divided into left and right hemispheres by sagittal incision. The left hemispheres were fixed in 4% paraformaldehyde (PFA) for the preparation of paraffin sections and subsequent immunohistochemical staining. Cortex and hippocampus tissues of the right hemispheres were dissected on ice, and immediately frozen in liquid nitrogen, and stored at −80°C for protein extraction and western blotting detection. A separate group pair of SF and NS mice (*n* = 3 for each group) were prepared for hippocampus dissection from fresh brain tissue and sent for RNA extraction and sequencing.

### Immunohistochemistry and Silver Staining

Immunohistochemistry was performed on 4 μm coronal paraffin-embedded sections. The sampled slices were deparaffinized and rehydrated in xylene and graded ethanol. Then, slices were placed in citrate antigen retrieval solution (PH 6) at 96°C for 20 min for heat-induced antigen retrieval. Slices were incubated with 3% H_2_O_2_ for 25 min to block endogenous peroxidase. Non-specific binding sites were blocked by 10% donkey serum for 1 h at room temperature. The slices were incubated with the following primary antibodies: mouse anti-Tau5 (dilution 1:400, ab80579, Abcam, UK), rabbit anti-p-Tau (Ser396) (dilution 1:200, ab109390, Abcam), rabbit anti-p-Tau (Thr231) (dilution 1:400, ab151559, Abcam), mouse anti-p-Tau (Ser202/Thr205) (AT8, dilution 1:200, MN1020, Invitrogen, USA), rabbit anti-Iba-1 (dilution 1:500, 019–19741, Wako, Japan), and mouse anti-GFAP (dilution 1:50, 3670, CST) overnight at 4°C. In order to detect the specific binding of primary antibodies, the horseradish peroxidase-conjugated appropriate secondary antibodies (Servicebio Inc., China) were applied for 1 h at room temperature. Antibody binding was visualized by reaction with diaminobenzidine. The slices with hematoxylin were counterstained and then dehydrated. The micrographs were quantified by a blinded investigator using the Image-Pro Plus software (MediaCybernetics, USA). Then, after deparaffinization and rehydration, slices were also conducted silver staining by using a silver staining kit (Servicebio Inc., China).

### Western Blotting

The cortex and hippocampus of the mice were homogenized in RIPA lysis buffer (Beyotime, China) with protease inhibitor cocktail and phosphatase inhibitor (Roche, Switzerland). Protein concentration was determined by using a BCA Protein Assay Kit (Beyotime, China). A total of 30 μg of protein per well was loaded on SDS–PAGE gels. Separated proteins on the gel were then transferred onto the PVDF membrane (0.22 μm, Millipore, USA) after electrophoresis. The PVDF membranes were blocked by 5% non-fat milk for 1 h at room temperature and then incubated with primary antibodies on a shaker overnight at 4°C. The following primary antibodies were used for Western blotting: mouse anti-Tau5 (dilution 1:1,000, ab80579, Abcam), rabbit anti-p-Tau (Ser396) (ab109390, Abcam), rabbit anti-p-Tau (Thr231) (dilution 1:1,000, ab151559, Abcam), mouse anti-p-Tau (Ser202/Thr205) (AT8, dilution 1:500, MN1020, Invitrogen), mouse anti-GFAP (dilution 1:1,000, 3670, CST), and mouse anti-β-actin (1:5,000, A5316, Sigma-Aldrich, USA). The membranes were incubated with appropriate HRP-conjugated secondary antibodies (Jackson ImmunoResearch, USA) for 1 h at room temperature. The bands were visualized by using enhanced chemiluminescence kits (Advansta, USA) *via* a Bio-Rad ChemiDoc XRS+ imaging system (USA). The gray values of the bands were analyzed by using ImageJ software.

### RNA Extraction and Sequencing

The total RNA of the hippocampus was extracted from NS and SF mice using Trizol (Invitrogen). The hippocampus was grounded into powder using liquid nitrogen, the appropriate volume of Trizol was added and homogenized for 2 min. Then, the content was rested for 5 min and centrifuged at 12,000 g for 5 min at 4°C. The supernatant was moved into a new EP tube, the appropriate volume of chloroform was added, and the tubes were shaked for 15 s, then the samples were left at room temperature for 2 min. The tubes were centrifugated for 15 min at 12,000 g, 4°C. After centrifugation, the upper aqueous phase containing RNA was moved into a new EP tube and isopropyl alcohol was added, the mixture was incubated at room temperature for 10 min. The contents were centrifuged at 12,000 g for 10 min at 4°C, then the supernatant was poured off and the pellet was kept. The pellet was washed with 75% ethanol and centrifuged again at 12,000 g for 5 min at 4°C. The supernatant was decanted into tubes and air-dried at room temperature for 5 min. The quality of these samples was verified, and mRNA library construction was performed by using Illumina Novaseq 6,000 by Shanghai Majorbio Bio-pharm Tech Co. Ltd. (China).

### Differential Expression Gene Analysis and Functional Annotations

Due to the limited sample size (*n* = 3 for each group), every sample of the SF group was paired with the 3 samples in the NS group to sort out the differential expression gene (DEG) lists. It gave rise to 9 pairs of comparisons. DEG analysis was performed by using the edgeR software (*p*-adjust < 0.05 and | log2Fold Change | ≥ 1). The DEGs present in more than 3 comparisons as the DEGs between NS and chronic SF group were listed. The functional annotation of DEGs by classification and enrichment analysis of the Gene Ontology (GO) and Kyoto Encyclopedia of Genes and Genomes (KEGG) was conducted.

### Comparisons of SF-Induced Hippocampal Transcriptome Characteristics With Other Disease Models

Through works of literature and GEO database search, the DEGs lists were extracted from the published data sources of mouse hippocampal RNAseq, including GSE168137 for Alzheimer's disease (5xFAD, congenic C57BL/6J background), GSE 61915 for normal aging (29 month old, C57BL/6J background), GSE140205 for APOEε4 mutation (humanized APOEε4 targeted replacement homozygous mice, C57BL/6J background), as well as the acute stress model (wild-type C57BL/6J background) (Pulga et al., 2016) and the stress model induced by hypergravity interventions (wild-type C57BL/6J background) (Pulga et al., 2016). The DEseq2 Software was used to sort out DEGs (*p*-adjust < 0.05; | log2FoldChange | ≥ 1). Five DEGs lists were generated in correspondence to the 5 models. Based on these DEGs lists, the expression levels (quantified as Fragments Per Kilobase per Million, FPKM) were extracted from the current hippocampal transcriptome data. Cluster analysis was performed on the 5 gene lists and the FPKM readouts of the data, respectively, and the relative expression levels of genes were displayed with heatmaps.

### Statistical Analysis

All quantities were characterized with mean and standard deviation calculated in the NS and SF groups. The comparison between these two classes was performed with a standard two-tailed *t*-test if the data qualified for normal distribution and homogeneity of variance, otherwise, with a Mann Whitney-test. Data were analyzed using GraphPad Prism 6. Differences were considered significant if *P* < 0.05.

## Results

### Chronic SF Increased Pathologically Phosphorylated Tau (Ser396) in Young Wild-Type Mice Brain

To investigate another set of important pathological proteins in AD, total tau, and phosphorylated tau(p-tau) at residues Thr231, Ser396, and Ser202/Thr205, we quantified tauopathy in the cortex and hippocampus of SF and NS mice. Western blot identified that the ratio of p-tau (Ser396)/total Tau5 was significantly higher in chronic SF hippocampus (SF vs. NS: 1.5 ± 0.42 vs. 1 ± 0.28, *n* = 9 for SF and *n* = 8 for NS, Unpaired *t*-test, *P* < 0.01). Although the ratio of p-tau (Ser396)/total Tau5 was higher in the SF cortex, it was not significant. And the ratio of p-tau (Thr231)/total Tau5 was also comparable between chronic SF and control group. Total Tau5 protein levels did not show a significant difference between both groups (Figures 1B,C). Immunohistochemistry staining was consistent with the results of western blot. Phosphorylated tau and p-tau (Ser396) exhibited a more condensed deposition in the cytoplasm of both cortex and hippocampus in the chronic SF group, while p-tau (Thr231) staining was similar between the SF group and control group (Figure 1D). However, phosphor-tau (Ser202/Thr205) was comparable between NS and SF groups. We further confirmed its expression by western blotting using AT8 antibody and found AT8 was almost absent in cortex samples of SF mice while it showed strong protein expression in the cortex of 12-month-old APP/PS1 mice (Supplementary Figure 1). Therefore, the chronic SF in young wild-type mice could increase certain pathological p-tau expressions in the hippocampus.

### Chronic SF Induced Gliosis in Both Cortex and Hippocampus

**Figure 2:**
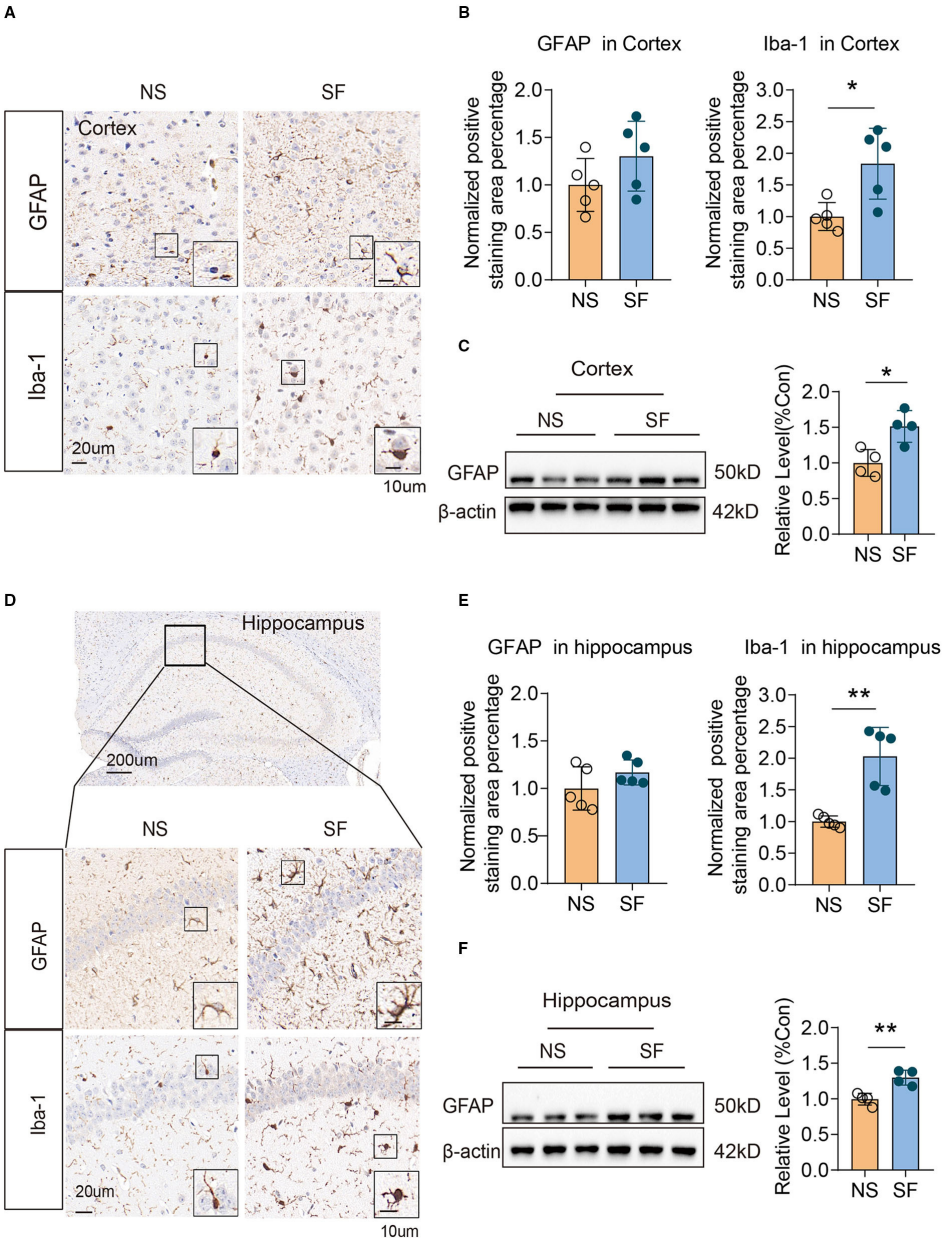
Chronic SF induces gliosis in the mouse cortex and hippocampus. **(A–C)** Activation of astrocyte and microglia in SF cortex. **(A)** Representative immunohistochemistry images of GAFP and Iba1 staining in the cortex of NS and SF mice. Scale Bar = 20 μm. Astrocytes labeled by GFAP, and microglia labeled by Iba1 were shown in the boxes. Scale Bar = 10 μm. **(B)** Quantitative analysis of positive staining of GFAP and Iba1 in the cortex of NS and SF mice. **(C)** Western blotting and quantitative analysis of GFAP in NS and SF cortex. **(D–F)** Activation of astrocyte and microglia in SF hippocampus. **(D)** Representative immunohistochemistry images of GFAP and Iba-1 staining in the hippocampus of NS and SF mice. A representative image of the mouse hippocampus was shown. Scale Bar = 200 μm. GFAP and Iba1 staining in the hippocampus CA1 region were shown in the enlarged images. Scale Bar = 20 μm. Astrocytes labeled by GFAP, and microglia labeled by Iba1 were shown in the boxes. Scale Bar = 10 μm. **(E)** Quantitative analysis of positive staining of GFAP and Iba1 in the hippocampus of NS and SF mice. **(F)** Western blotting and quantitative analysis of GFAP in NS and SF hippocampus. For immunohistochemistry, *n* = 5; for western blotting *n* = 4 per group. *P < 0.05, **P < 0.01.

Gliosis was evident in a variety of pathological conditions, which links tightly with injury repair, neuroinflammation. The positive staining area of microglial marker (Ionized calcium-binding adapter molecule 1, Iba-1) Iba-1, were both significantly higher in chronic SF group vs. control (cortex: 1.84 ± 0.56 vs. 1 ± 0.22, *n* = 5, Unpaired *t*-test, *P* < 0.05; hippocampus: 2.03 ± 0.46 vs. 1 ± 0.09, *n* = 5, Unpaired *t*-test, *P* < 0.01) (Figures 2A,B,D,E). As for quantification of the astrocytic marker (glial fibrillary acidic protein, GFAP) expression, western blot of GFAP protein was significantly higher in the chronic SF group in both cortex (SF vs. NS: 1.51 ± 0.22 vs. 1 ± 0.19, *n* = 4, Unpaired *t*-test, *P* < 0.05) (Figure 2C) and hippocampus (SF vs. NS: 1.3 ± 0.1 vs. 1 ± 0.08, *n* = 4, Unpaired *t-*test, *P* < 0.01) (Figure 2F), while the positive area of GFAP labeled by immunochemistry staining did not reach a statistically significant difference (Figures 2A,B,D,E). These results were consistent with our previous data (Xie et al., 2020a) and characterized the activation of gliosis in both cortex and hippocampus in the chronic SF group.

### Chronic SF Enhanced Glucose Metabolism in Multiple Brain Regions

**Figure 3:**
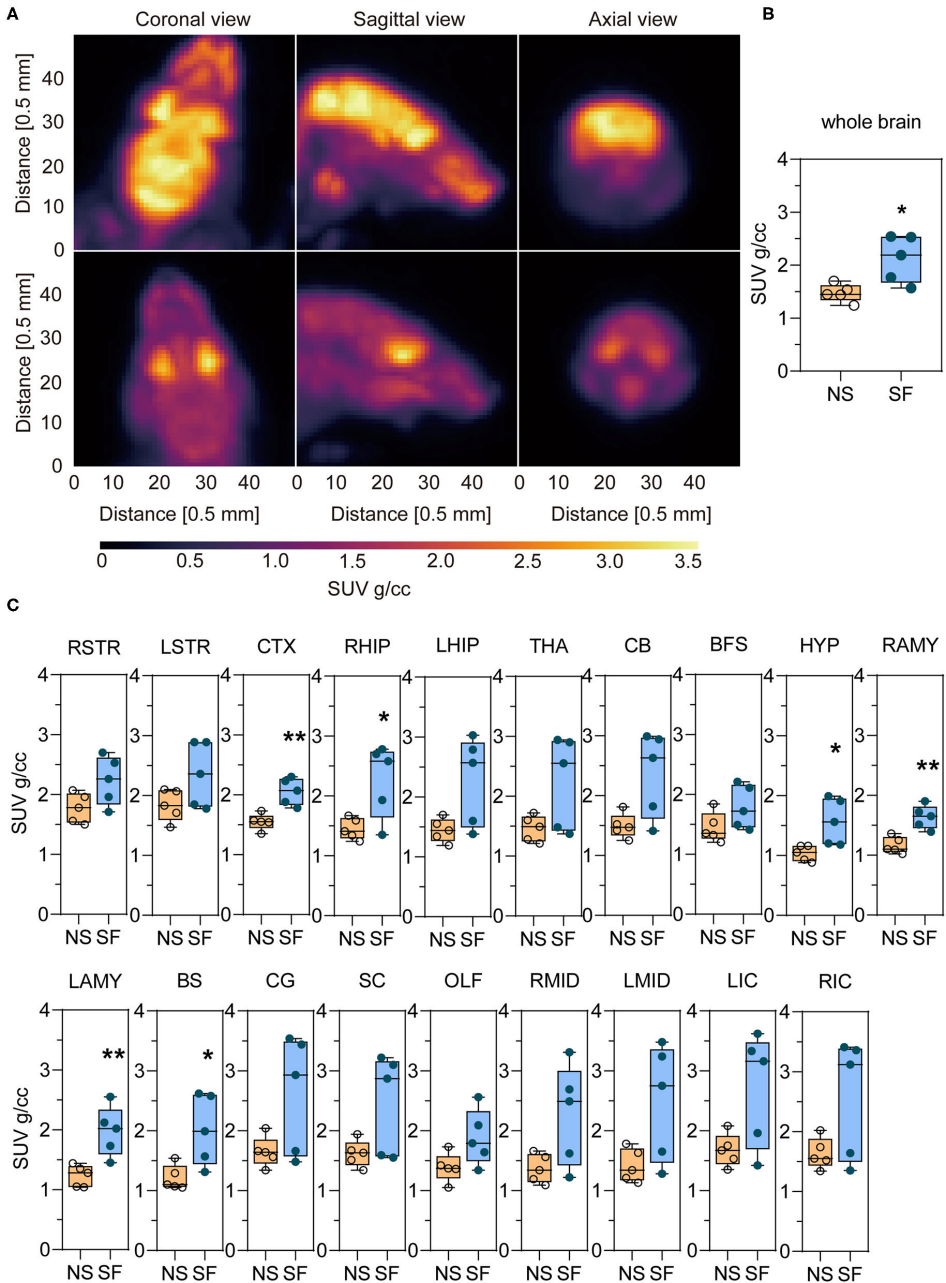
Chronic SF enhances the glucose uptake in the brain monitored by ^18^F-FDG-PET/CT. **(A)** Coronal, sagittal, and axial view of the brain images of an SF (top) and NS (bottom) mouse. **(B)** Statistical analysis of whole-brain SUV in NS and SF group. *n* = 5 per group. *P < 0.05. **(C)** ^18^F-FDG uptake in NS and SF mice in different brain regions expressed as SUV. *n* = 5 per group. *P < 0.05, **P < 0.01. See Table 1 for the nomenclature of the brain regions.

An example of the ^18^F-FDG PET image of an SF and NS mouse is shown in Figure 3A. For a quantitative interpretation of the PET measurement, we summarized the SUV of each brain region, as well as the total SUV for both groups in Table 1. The chronic SF group exhibited a total SUV in the entire brain ~44% higher with respect to the NS group (Figure 3B) (Unpaired *t*-test, *P* < 0.05). More interestingly, the average SUV in all the 19 brain regions separately was higher in the SF group than in the NS group (Figure 3C). In particular, the increased glucose metabolism of the SF group was statistically significant in the cortex (+32%, Unpaired *t*-test, *P* < 0.01), right hippocampal region (+57%, Unpaired *t-*test, *P* < 0.05), Hypothalamus (+51%, Unpaired *t*-test, *P* < 0.05), right amygdala (+41%, Unpaired *t-*test, *P* < 0.01), left amygdala (+60%, Unpaired *t*-test, *P* < 0.01) and brain stem (+66%, Unpaired *t*-test, *P* < 0.05).

### Hippocampal RNAseq Revealed SF-Induced Alterations in a Broad Range of Pathways

**Figure 4:**
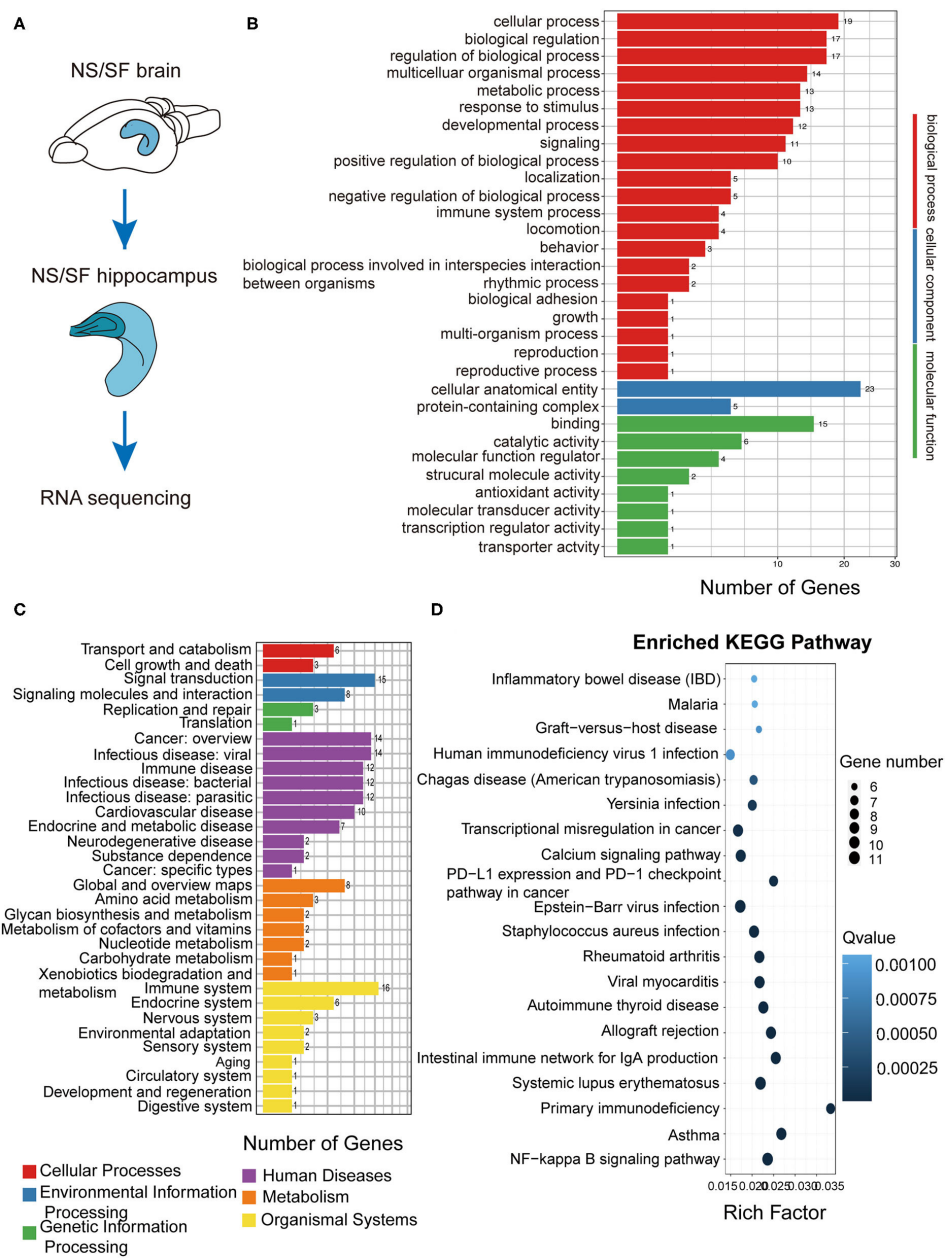
Functional annotation of DEGs from the hippocampus in SF mice. **(A)** The schematic of hippocampus RNA sequencing. **(B)** The differential gene GO function classification map of the hippocampus. **(C)** KEGG classification on the DEGs from the SF hippocampus. **(D)** Statistics of KEGG pathway enrichment of DEGs map of SF hippocampus. The X-axis represents the enrichment factor, the Y-axis represents the pathway name, and color represents the Q value; the smaller the value, the more significant the enrichment result; the size of the point represents the number of DEGs. *n* = 3 per group.

Hippocampus is the key region involved in learning and memory. To explore the molecular alterations induced by chronic SF, we compared the RNA sequencing in hippocampal tissue between the two groups (Figure 4A). We sorted out a list of genes as DEGs following bioinformatics analysis flow and we found 98 DEGs genes (see Supplementary Table 1). We further conducted GO, KEGG biological pathway classification, and enrichment analysis. GO terms with enriched gene numbers are shown in Figure 4B, the highlighted biological processes included metabolic process, response to stimulus, immune system process, locomotion, etc. KEGG biological pathway classification is shown in Figure 4C, where several pathways involving inflammation, immune system, metabolic functions, aging, cancer, are visible. Enriched KEGG pathways are listed in Figure 4D. They involve infectious diseases, autoimmune diseases, allergic diseases, transcriptional misregulation in cancer, and calcium signaling pathway, NF-κB signaling pathway. These results found a broad range of biological processes involved in the pathogenesis in the hippocampus induced by chronic SF, mostly linking with immune system dysfunction, inflammation, metabolic dysregulation, and molecular transcription misregulation.

### SF-Induced Transcriptome Alterations Were Similar With Stress Models But Not With Neurodegeneration or Aging Models

**Figure 5:**
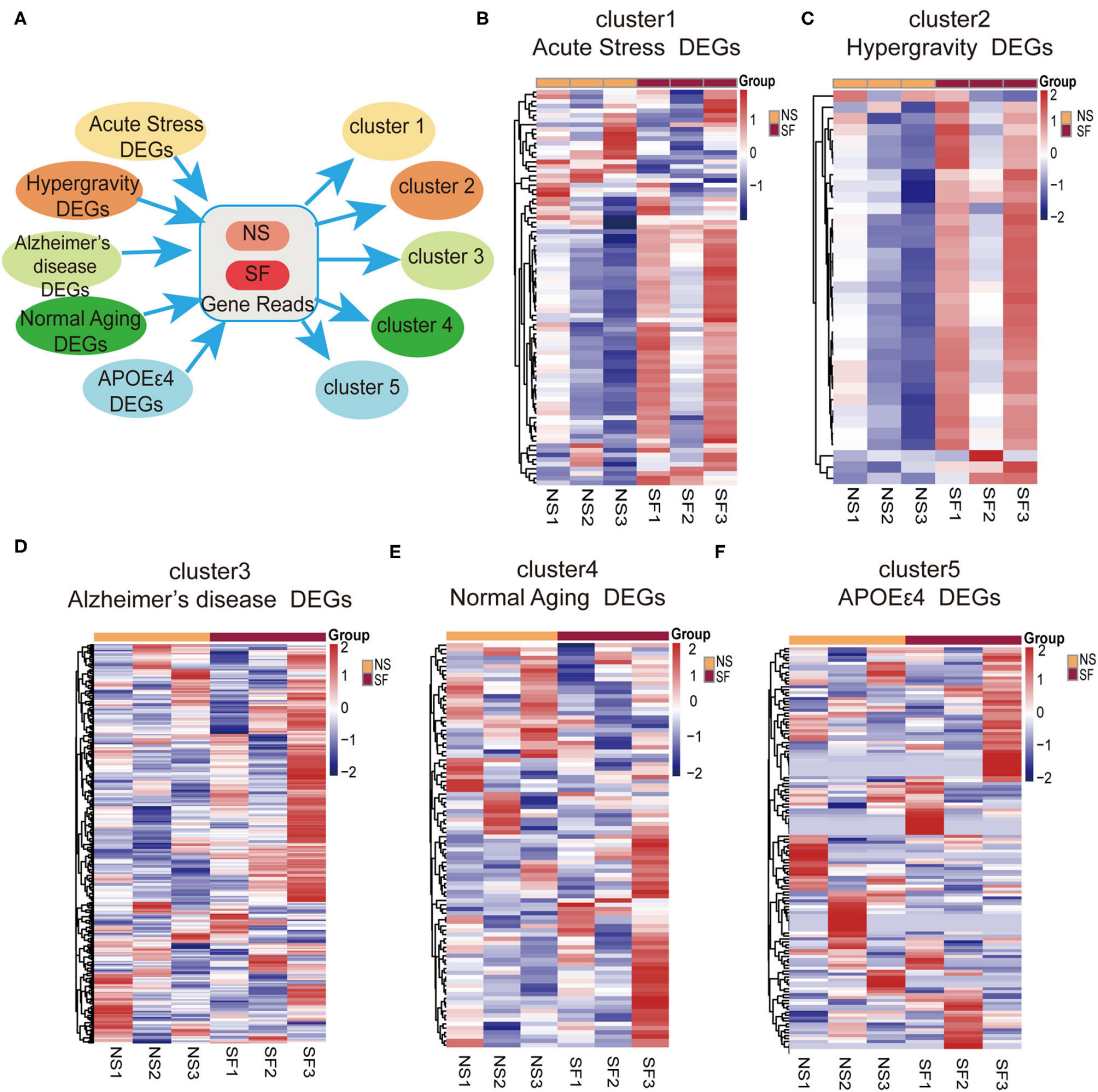
Bioinformatic analysis revealed a link between chronic SF and stress. **(A)** Schematic instruction of cluster analysis of SF and related diseases or conditions. **(B–F)** Heat map showing relative gene expression in SF involved in different conditions [**(B)** acute stress, **(C)** hypergravity, **(D)** Alzheimers disease, **(E)** normal aging, **(F)** APOEε4] *n* = 3 per group.

In order to better understand the pathological processes up- or down-regulated by chronic SF, we plotted the expression patterns of the DEGs extracted from hippocampal RNAseq experiments of several disease models with cluster analysis. First, we found the DEG lists from the published hippocampal RNA sequencing data in mouse models of Alzheimer's disease model (5xFAD on congenic C57BL/6J background, GSE168137), normal aging model (29-month-old C57BL/6J mice, GSE 61915), APOEε4 mutation model (humanized APOEε4 targeted replacement homozygous mice on C57BL/6J background, GSE140205), as well as two types of stress models (wild-type C57BL/6J background) (Pulga et al., 2016). Five DEG lists were generated corresponding to these 5 disease models. Based on these 5 gene lists, the expression levels (FPKM) were extracted from our current hippocampal transcriptome data. Cluster analysis was performed based on the 5 gene lists and the FPKM readouts, respectively (Figure 5A). The clustering heatmap demonstrates, that the gene lists of both stress models exhibit clear differential expression patterns between SF and NS mice (Figures 5B,C). One of the stress models was induced by intraperitoneal injection of 10 mg corticosterone, the other stress models were obtained by placing the mice under a hypergravity condition of 3G for 21 days (Pulga et al., 2016). As visible in the clustering heatmaps corresponding to the gene lists of Alzheimer's disease, normal aging, and APOEε4 mutation model, there are no significant differential expression patterns between SF and NS groups (Figures 5D–F). In other words, the major hippocampal transcriptome alterations induced by those 3 models were mostly not present in the chronic SF model. This analysis showed that SF-induced transcriptome alterations were similar with stress models but not with models of neurodegeneration or normal aging.

## Discussion

We have been interested in answering the scientific question: does chronic sleep fragmentation leads to Alzheimer's disease even in mice without genetic pre-disposition and senescence? Based on our previous study, we found intracellular Aβ_1−42_ accumulation and protein degradation pathway dysfunction, as well as obvious cognitive decline in young wild-type mice after 1.5-month sleep fragmentation (Xie et al., 2020a). However, it was largely unknown what pathological processes were occurring in the chronic SF brain, and whether they provided the preceding conditions for developing neurodegeneration pathogenesis. Herein, as a follow-up study, we further found 1.5-month chronic SF could induce intracellular accumulation of pathological hyperphosphorylated tau (Ser396) and gliosis in young wild-type mice brains (Figures 1, 2). ^18^F-FDG PET scan identified a distinctive feature of chronic SF, namely the brain glucose utilization was upregulated, indicating an elevated metabolic activity throughout brain regions (Figure 3). Hippocampal RNA sequencing further showed that chronic SF induced alterations in a broad range of pathways, involving the immune system, inflammation, and metabolic dysregulation, transcriptional misregulation (Figure 4). In combination with 5 other models available hippocampal RNA seq data, we observed the stress models resulted in similar alterations of hippocampal transcriptome characteristics with chronic SF. To our surprise, the typical transcriptome changes in Alzheimer's disease, normal aging, and APOEε4 mutation were almost not present in chronic SF (Figure 5). Thus, 1.5-month SF in young wild-type mice induced pathological processes which were similar to stress models, but not with neurodegenerative disease and senescence models. However, current data indicated that, even without genetic background and senescence, the ongoing pathological processes in sleep fragmented brain provided the preceding basis for developing neurodegeneration if the stressor continuously exits. We will discuss it from several perspectives below.

### Chronic Sleep Fragmentation Resulted in Neurotoxic Protein Accumulation

The two main pathological hallmarks of AD are senile plaques composed of amyloid-β (Aβ) and neurofibrillary tangles (NFTs) comprised of hyperphosphorylated tau. Clinical evidence found that cognitively normal individuals with amyloid-β deposition showed worse sleep quality compared with those without amyloid deposition, meanwhile, Aβ plaques could occur 10–15 years before cognitive impairment in AD (Ju et al., 2013). It matches our previous observation of intracellular Aβ_1−42_ in sleep fragmented brain (Xie et al., 2020a). The “intracellular amyloid hypothesis” suggested that neuronal necrosis occurs in the ultra-early stage of Alzheimer's disease due to intracellular amyloid accumulation. Okazawa et al. explained that the early accumulation of intracellular amyloid deprives a critical effector molecule, Yes-associated protein (YAP) in the Hippo signaling pathway that is essential for cell survival (Tanaka et al., 2020). The initial neuronal necrosis releases high mobility group box 1 (HMGB1) into the interstitial space and induces a cluster of secondary necrosis in the surrounding area (Okazawa, 2021). Okazawa et al. also reported that inhibition of HMGB1 by anti-HMGB1 antibody prevents progression of neurodegeneration (Fujita et al., 2016). These studies supported the hypothesis that interstitial space Aβ deposition (senile plaques) doesn't represent the peak of pathological cascade since the pathological processes were initiated much earlier at the stage of intracellular amyloid accumulation. The significantly increased ratio of pathological hyperphosphorylated tau (Ser396)/total tau found in sleep fragmented brains could be partially explained by intracellular protein degradation dysregulation. We previously reported the aberrant expressions of critical molecules for endosome-autophagosome-lysosomal (EAL) pathways (Xie et al., 2020a). Therefore, intracellular AD-like neurotoxic accumulation could be initiated in young healthy brains due to chronic sleep fragmentation.

### Disrupted Normal Circadian Rhythms Induced Neuroendocrinological Dysregulation and Emotional Stress

It has been proposed that emotional stress, mainly including anxiety and depression, links with cognitive decline and AD pathogenesis (Mendez, 2021). Furthermore, according to the observation, stress disorders, such as PTSD, were associated with the development of dementia (Bonanni et al., 2018). The aged primates having a bad early life experience showed more amyloid plaque deposition in the neocortex and a significant reduction in synaptophysin, suggesting stress increased the vulnerability to neurodegeneration (Merrill et al., 2011). We previously identified both cognitive decline and anxiety-like behaviors in 1.5-month SF mice (Xie et al., 2020a). The hypothalamus-pituitary-adrenal (HPA) axis responds to stress, inducing the adrenal cortex release of glucocorticoid hormones, cortisol, in humans. In patients with AD, high basal cortisol concentration is associated with smaller hippocampus volume and cognitive decline (Huang et al., 2009). Similarly, high cortisol levels in plasma and cerebrospinal fluid (CSF) are found related to the progression of cognition decline, which is useful in predicting clinical worsening from MCI to AD (Popp et al., 2015; Lehallier et al., 2016). Glucocorticoid was reported to increase the expression of amyloid precursor protein (APP) and β-site APP-cleaving enzyme1(BACE1) in astrocytes, and decrease some Aβ-degrading proteases, leading to Aβ deposition (Bai et al., 2011). Moreover, hippocampal corticosteroid exposure can promote tau phosphorylation *via* activating glycogen synthase kinase 3β(GSK3β) (Dey et al., 2017). In addition, glucocorticoids impair the endo-lysosomal and autophagic mechanisms of tau degradation, inducing accumulation of aggregated tau (Vaz-Silva et al., 2018; Silva et al., 2019).

Sleep has a suppressive effect on the HPA stress system. Sleep disturbance would maintain or further elevate the activity of the stress system, which could affect the circadian rhythm of stress system functional dynamics. Studies have shown that acute partial sleep loss can alter glucocorticoid regulation, increasing the cortisol level the next evening (Leproult et al., 1997). In the current study, we found out that differential expression gene lists from two stress mice models showed differential expression patterns between 1.5-month SF and control mice, while Alzheimer's disease, normal aging, and APOEε4 mutation models did not exhibit any significant differential expression patterns (Figure 5). One of the stress models was induced by intraperitoneal injection of corticosteroid to mimic acute stress, while the other model was to give continuous hypergravity stress during certain time blocks for 21 days. The later model displayed the best clustering pattern (Figure 5), which also shared the most similarity with our chronic sleep fragmentation models. In summary, chronic sleep fragmentation in young wild-type mice induced neuroendocrinology system dysregulation and hippocampal transcriptome alterations similar to stress models. It provided supporting evidence for the tight connections observed clinical-wise between emotional stress and AD pathogenesis.

### Chronic Sleep Fragmentation Induced Brain Glucose Metabolism Imbalance

In the central nervous system, glucose metabolic rate reflects neuronal activity, synaptic density, and neuroinflammation. ^18^F-FDG, the most commonly used radiotracer analog of brain glucose, was used to capture the brain glucose metabolic changes using a pre-clinical PET system. A previous study identified in APP/PS1 mice that, Tg mice of 2-month-old (equivalent to pre-clinical) and 3.5-month-old (equivalent to sub-clinical) exhibited significant glucose utilization increase in multiple brain regions vs. normal controls. It was explained by a compensatory state due to neuronal hyperexcitability in pre- or early AD. In 2- and 3.5-month-old Tg mice, the hyperglycolytic brain regions were highlighted to be the entorhinal cortex, hippocampus, and frontal cortex (Li et al., 2016). It was proposed that hypermetabolism before the onset of clinical dementia could be an early biomarker for AD diagnosis in clinical patients (Herholz, 2010). So far as we know, we were the first to characterize brain glucose metabolism in a chronic sleep fragmentation model with ^18^F-FDG-PET in young wild-type mice. We found out that the SUV values in brain regions of the cortex, hippocampus, hypothalamus, amygdala, brain stem were significantly enhanced in the SF group (Figure 3; Table 1). Hippocampal pathology in AD patients usually happens early and contributes to cognitive dysfunction (Wang, 2014). Similar to APP/PS1 mice at the age of 2–3.5 month-old, hippocampus glucose metabolism in the SF brain was also increased, it matched with the cognitive decline in behavioral tests reported previously (Xie et al., 2020a). Meanwhile, the hypothalamus, hippocampus, amygdala, and brain stem link with the HPA axis (Fries et al., 2009; Contoreggi, 2015). Hypothalamus is the center regulating the stress hormone released from the pituitary and control of the autonomic nervous system *via* releasing corticotropin-releasing hormone (CRH) from the paraventricular nucleus (PVN). Amygdala has abundant CRH receptors, and it provides excitatory input to PVN and receives excitatory signals from PVN. Hippocampus also has high concentrations of corticosteroid receptors and has been implicated in negative feedback regulation of HPA axis activity. That explains why these regions were found closely linked with stress-related diseases, such as anxiety disorder, PTSD, autoimmune conditions, and metabolic syndrome (Contoreggi, 2015). It is consistent with the findings of anxiety-like behaviors alterations (Xie et al., 2020a) and stress-like hippocampal transcriptome changes in 1.5-month SF mice (Figures 4, 5). Alternatively, studies showed that neuronal synaptic strength/connectivity is higher during wakefulness than during sleep (Vyazovskiy et al., 2008; Bero et al., 2011). The enhanced glucose metabolism could also possibly be due to the disrupted circadian rhythms in SF mice, and all the PET scans were performed during the light-ON phase which is sleep hours for the NS group. In summary, the enhanced brain glucose metabolism could be due to hyperexcitability of neurons and networks, very similarly with pre- and early stages of AD. This explains the cognitive and emotional behavioral outcomes of SF mice from the perspective of energy imbalance and matches with stress-like hippocampal transcriptome changes.

### Chronic Sleep Fragmentation Induced Pathological Neuroinflammation in the Brain

Hippocampal RNAseq in 1.5-month SF and NS mice revealed that inflammatory and immunological pathways were activated by SF (Figure 4). Enrichment of the KEGG pathway showed that the signaling pathways involved in the DEGs were also mainly in infection (virus, malaria, and bacterial infection), immune-related pathways (IBD, rheumatoid arthritis, viral myocarditis, autoimmune thyroid disease, SLE, asthma, and NF-κB pathway) (Figure 4). Sleep deficiency can clearly induce systemic inflammation which links with the development of cardiovascular disease, autoimmune and neurodegenerative diseases (Besedovsky et al., 2019; Irwin and Vitiello, 2019). Inside the central nervous system, microglia function as macrophages in peripheral tissue, monitoring the pathogens and maintaining homeostasis in the brain. They are sensitive to pathological protein aggregates. Microgliosis has been widely observed in the pre- or early stages of neurodegenerative diseases. Their important contribution to neurodegeneration pathogenesis has been intensively studied (Thawkar and Kaur, 2019). We found that SF induced significant microgliosis both in the cortex and hippocampus (Figure 2). Interstitial fluid (ISF) Aβ and tau levels exhibit diurnal fluctuation, negatively linked with sleep time (Kang et al., 2009; Holth et al., 2019). Sleep deprivation increased overnight CSF Aβ_1−42_ levels in healthy adults (Ooms et al., 2014; Lucey et al., 2018). Worse sleep quality and slow-wave sleep (SWS) disruption are significantly associated with higher CSF tau levels (Ju et al., 2017; Holth et al., 2019). Therefore, the interstitial space microenvironment in SF could be quite similar to pre- or early stages of neurodegenerative diseases. The possible explanation could be that microglia were activated by SF-induced excessive interstitial Aβ, p-tau, dysregulated glucocorticoids levels (Fonken et al., 2016), and stress-induced by disrupted circadian rhythms (Fonken et al., 2015, 2016). We are wondering if these activated microglia could initiate neurodegenerative pathogenesis in the SF brain. Besides secretion of inflammatory cytokines which could directly harm the neuronal cells, microglia was reported to induce synapse elimination *via* C1q and C3 (Schafer et al., 2012), while C3 is upregulated after acute and chronic sleep deprivation (Bellesi et al., 2017). Meanwhile, activated microglia exhibiting amoeboid phenotype, termed as “primed” microglia, showed impaired phagocytic function and therefore dampened neurotoxins clearance (Norden et al., 2015). Thus, SF-activated microglia could probably undergo similar pathological processes as in neurodegeneration, since the stressors were quite similar in the pre- and early stages of neurodegenerative diseases, such as elevated neuroendocrine hormones, excessive metabolic waste, as well as cell debris generated from neuronal necrosis.

## Conclusion

The current study reported 1.5-month SF in young wild-type mice could generate AD-like pathological changes. Based on the hippocampal transcriptome analysis and literature evidence, we proposed that 1.5-month SF induced pathological processes which were quite similar with pre- and early stages of neurodegenerative diseases, however not yet reaching the symptomatic stage. The mechanisms shared in common were probably neuroinflammation, neuroendocrinological dysregulation, energy metabolism imbalance. Avoiding long-term sleep disturbance or dealing with chronic insomnia-induced stress could be a preventative strategy for neurodegenerative diseases.

## Data Availability Statement

The datasets presented in this study can be found in online repositories. The names of the repository/repositories and accession number(s) can be found below: https://www.ncbi.nlm.nih.gov/sra/PRJNA757396.

## Ethics Statement

The animal study was reviewed and approved by the Institutional Animal Care and Use Committee of Tongji Hospital, Tongji Medical College, Huazhong University of Science and Technology.

## Author Contributions

ND'A and FD designed the research and contributed to revisions and the final draft of the manuscript. LB and LH conducted the experiment, data analysis, and drafted the manuscript. YX contributed to brain sample preparations for RNA sequencing. ZHe participated in the bioinformatic analysis. SD contributed to setting up the chronic sleep fragmentation system. MZ, ZHu, and CY contributed to revisions. EA, WX, YL, and QX were responsible for the PET scans. All authors agree to be accountable for the content of the work.

## Funding

This work was supported by the National Natural Science Foundation of China (81801318) and Shanghai Scientific Society (No. 20ZR1403500; General project), in part by the MAECI Great Relevance 2019 contributions Italy-China (Grant No. PGR00846), in part by the National R&D Program for Major Research Instruments of Natural Science Foundation of China (6027808), in part by the National Key Research and Development of China (2019YFC0118900).

## Conflict of Interest

The authors declare that the research was conducted in the absence of any commercial or financial relationships that could be construed as a potential conflict of interest.

## Publisher's Note

All claims expressed in this article are solely those of the authors and do not necessarily represent those of their affiliated organizations, or those of the publisher, the editors and the reviewers. Any product that may be evaluated in this article, or claim that may be made by its manufacturer, is not guaranteed or endorsed by the publisher.

## Supplementary Material

Supplementary Figure 1 Phosphor-tau (Ser202/Thr205) is comparable between NS and SF. **(A)** Immunohistochemistry of phosphor-tau (Ser202/Thr205) with AT8 antibody in cortex and hippocampus in slices of NS and SF groups. Scale bar = 20 μm. **(B)** Western blot of AT8 in SF cortex, using cortex of 12-month-old APP/PS1 mice as the positive control. **(C)** Silver staining of brain slices collected from cortex and hippocampus of mice in NS and SF groups as well as APP/PS1 mice. Scale bar = 20 μm.
