# Supplementary figures and images for: Does Chronic Sleep Fragmentation Lead to Alzheimer's Disease in Young Wild-Type Mice?
Source: Front Aging Neurosci. 2021 Dec 21;13:759983. doi: 10.3389/fnagi.2021.759983 (PMC8724697; doi:10.3389/fnagi.2021.759983)

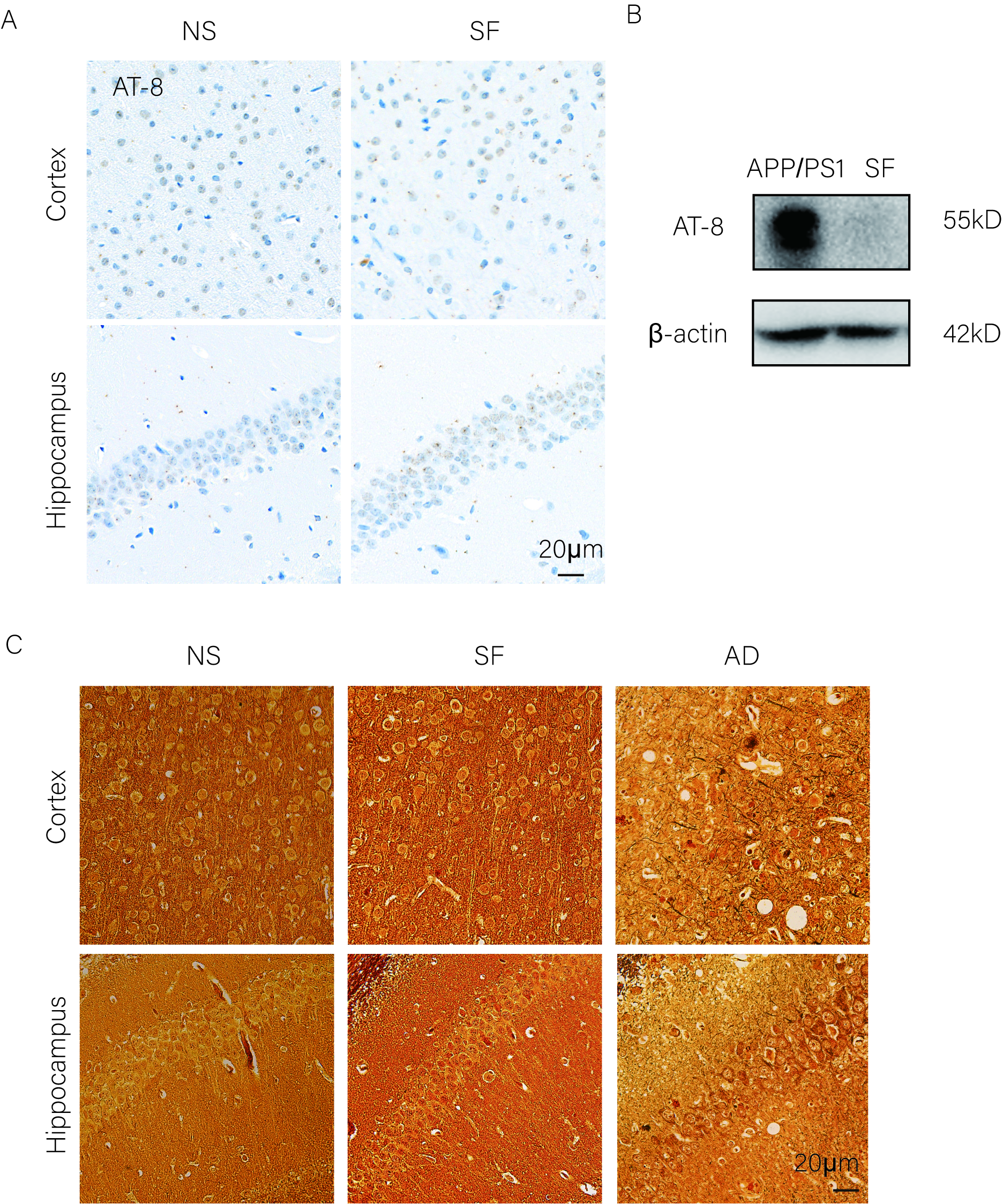

Supplement: Supplementary Figure 1 — Phosphor-tau (Ser202/Thr205) is comparable between NS and SF. (A) Immunohistochemistry of phosphor-tau (Ser202/Thr205) with AT8 antibody in cortex and hippocampus in slices of NS and SF groups. Scale bar = 20 μm. (B) Western blot of AT8 in SF cortex, using cortex of 12-month-old APP/PS1 mice as the positive control. (C) Silver staining of brain slices collected from cortex and hippocampus of mice in NS and SF groups as well as APP/PS1 mice. Scale bar = 20 μm. [file Image_1.TIF]
